# Craniofacial morphology of wind and string instrument players: a cephalometric study

**DOI:** 10.1186/s12880-020-00455-6

**Published:** 2020-05-26

**Authors:** Miguel Pais Clemente, Joaquim Mendes, André Moreira, Afonso Pinhão Ferreira, José Manuel Amarante

**Affiliations:** 1 Department of Surgery, Faculty of Medicine, INEGI, Labiomep, University of Porto, Alameda Prof. Hernâni Monteiro, 4200-319, Porto, Portugal; 2 Department of Mechanics, Faculty of Engineering, University of Porto, R. Dr. Roberto Frias, 4200-465, Porto, Portugal; 3 Speacialization Student in Oral Rehabilitation, Faculty of Dental Medicine, University of Porto, 392, R. Dr. Manuel Pereira da Silva, Porto, Portugal; 4 Department of Orthodontics, Faculty of Dental Medicine, University of Porto, 392, R. Dr. Manuel Pereira da Silva, Porto, Portugal

**Keywords:** Cephalometric, Craniofacial morphology, Malocclusion, Musicians, String wind instrumentalists

## Abstract

**Background:**

Playing an instrument may promote a parafunctional behavior within the cranio-cervical-mandibular-complex with unknown repercussions. The aim of this study was to find any association between the dental inter-arch relationship and the practice of a wind or string instrument.

**Methods:**

A sample of 77 musicians, divided in two groups of wind (*n* = 50) and string instrumentalists (*n* = 27), had a lateral cephalogram taken to compare six cephalometric parameters following the Rickett’s analysis (maxilla position, mandible position, facial type, skeletal class, upper incisor and lower incisor inclination). The Fisher test was performed to compare, with a 95% statistical confidence, if both groups have similar frequency distributions for each cephalometric parameter.

**Results:**

No statistical differences were found for the maxilla position, mandible position, facial type, skeletal class and upper incisor inclination. Statistical differences were found for the lower incisor inclination (*p* = 0.011).

**Conclusions:**

Playing a wind instrument showed to have little orthopaedic influence at the craniofacial morphology, on contrary it may influence the lower incisor inclination with its osseous base.

## Background

Craniofacial morphology has been associated to distinct aspects such as genetic factors and the influence of the local environmental factors, where the activity and function of the masticatory system is important [1]. Some authors suggest a relationship between craniofacial morphology and the force or structure of the masseter muscles [1–6]. In some cases a correlation has been found between the size and morphology of the jaw, more precisely the height, the length of the mandibular ramus and the angle of the mandible, with the bite force resulting from the primary activity of the masseter muscles [2, 7]. Nevertheless, only few researchers have attempted to explain the interrelationship of craniofacial morphology and bite force described by cephalometric analysis [1, 4, 5, 8].

If one takes in consideration that the masticatory system is part of a complex neuromuscular system that coordinates different tasks, which primary functions are mastication, swallowing and speech, and the secondary functions are breathing and the expression of emotions. For this purpose one should bear in mind that the activity of playing a wind or a string instrument can promote a parafunction within the cranio-cervical-mandibular-complex (CCMC), since playing a saxophone means that there will require an isometric contraction of the masseter muscle while stabilizing the mouthpiece within the orofacial structures. In the same way, a string instrument like for example a violin requires an asymmetrical pattern and activity of postural muscles such as the sternocleidomastoid muscle during musical performance. Beyond this, during the stabilization of the violin, there will probably be an hyperfunction of the elevator muscles of the masticatory system, since it is common to clench his teeth while holding the instrument between the angle of the mandible, the shoulder and the chest [9].

The central nervous system coordinates these different functions of the stomatognathic system, which in some cases can be altered by several factors, such as dental occlusion, neuromuscular dysfunction, periodontal mechanoreceptors and pain [10].

Some attempts have been made in the past in order to associate musical performance and an eventual effect on modifying facial morphology when playing violin and viola [11]. On another research [12], a cross sectional observational study was carried out comparing the occlusions of 170 professional musicians, where the impressions of the teeth of each subject and the occlusal parameters were assessed from the dental casts. The authors concluded that playing a wind instrument does not significantly influence the position of the anterior teeth and is not a major etiologic factor in the development of malocclusion [12]. Rindischeber et al., analysed 62 professional musicians wind instrument players, 31 brass instrument players and 31 wind instrumentalists composed by the reed and flute instrument group. There were no significant differences between the two groups of musicians, or between the reed instrument group and the control group [13]. Understanding the influence of musical instruments on tooth positions has been the aim of some studies, Herman carried out a 2-year longitudinal investigation at five New York City junior schools on 11 to 13 year old children, which presented statistically significant anterior tooth movements in the majority of the instrumentalists when compared to the control group [14]. José Gloria et al., also found that within a sample of 40 professional musicians who played wind instruments, mainly saxophone, clarinet and flute, the main oral change that affected them was the inclination of anterior teeth [15]. In a systematic review conducted by van der Weijden et al., 2018 the authors propose that tooth position can influence a wind instrumentalist’s performance and affect the comfort of its embouchure [16]. There has been an association between the respiratory pattern and craniofacial morphology [17–19], were for example the inter-arch growth pattern can be influenced by an unbalanced muscular function of mouth breathers [20]. These individuals with nasal respiratory dysfunction can have maxilla-facial discrepancies like “adenoid faces” [21], the presence of open bite [8, 10], a reduced transversal dimension with a high arched palate [22, 23] associated or not with a posterior crossbite and the presence of a class II malocclusion [24–26].

Therefore it would be interesting to study if there is any association between the dental inter-arch relationship and the practice of a specific wind or string instrument. The purpose of this study is to compare six cephalometric parameters between wind and string instrumentalists.

## Methods

### Sample

The present study was composed by a sample of 77 participants, 50 wind instrumentalists (*n* = 50; 64.9%) and 27 string instrumentalists (*n* = 27; 35.1%). The majority of musicians were associated to the orchestra national of Porto, Casa da Música, and the students from a Master of Science degree in Music and Performing arts from Superior School of Music and Performing Arts of Oporto (ESMAE). The wind instrumentalists were clarinetists (*n* = 10), saxophonists (*n* = 8), bassoon players (*n* = 4), tuba players (*n* = 4), trumpeters (*n* = 10), oboists (*n* = 1), french horn players (*n* = 4), trombone players (*n* = 4) and flautists (*n* = 5). The string instrumentalists were violinists (*n* = 14), violoncellists (*n* = 6), viola players (*n* = 4), contrabass players (*n* = 2) and one pianist. The inclusion criteria were adults (> 18 years old), with more than 10 years of experience and no prior orthodontic treatment nor any history of maxillofacial surgery neither mandibular injuries.

### Ethical approval

The present study has been in accordance with the World Medical Association Declaration of Helsinki. Moreover, the research was approved by the ethics committee of Faculty of Dental Medicine, University of Porto, no. 880292. A written consent was obtained from all participants involved in this study explaining the objective of the study and its methods.

### Craniofacial morphology

**Fig. 1:**
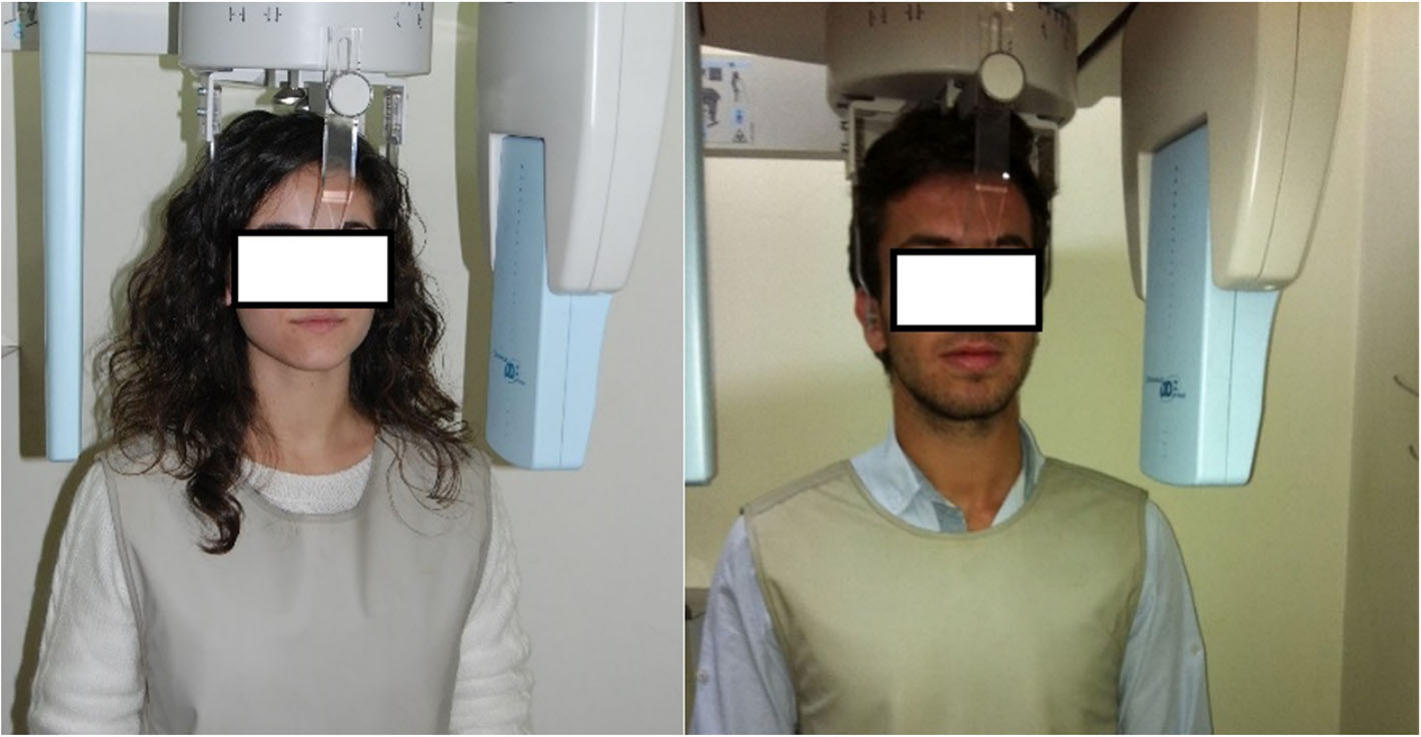
Lateral cephalogram acquisition of a string instrumentalist and a wind instrumentalist

The present research uses lateral cephalograms to determine craniofacial morphology. The lateral cephalograms were taken at the Faculdade de Medicina Dentária da Universidade do Porto (Fig. 1) with the Orthoralix 9200 - Gendex, KaVo, Biberach an der RiB, Germany. The images were taken to both, string and wind instrumentalists, in maximum intercuspidation by the same technician to allow a standardization of the protocol.

The participants were told to not move during the imaging acquisition, look forward, feet aligned, head in rest position, but in the orthostatic position. It was told to the participants to hold their head in a stabilized position with the olives in external auditory meatus and with the indicator supporting the glabella. The subject’s sagittal plane was perpendicular to the path of the x-ray. The Frankfort (horizontal) plane was parallel to the floor. To all participants was given a lead vest to minimize the radiation exposure.

### Data and statistical analysis

**Fig. 2:**
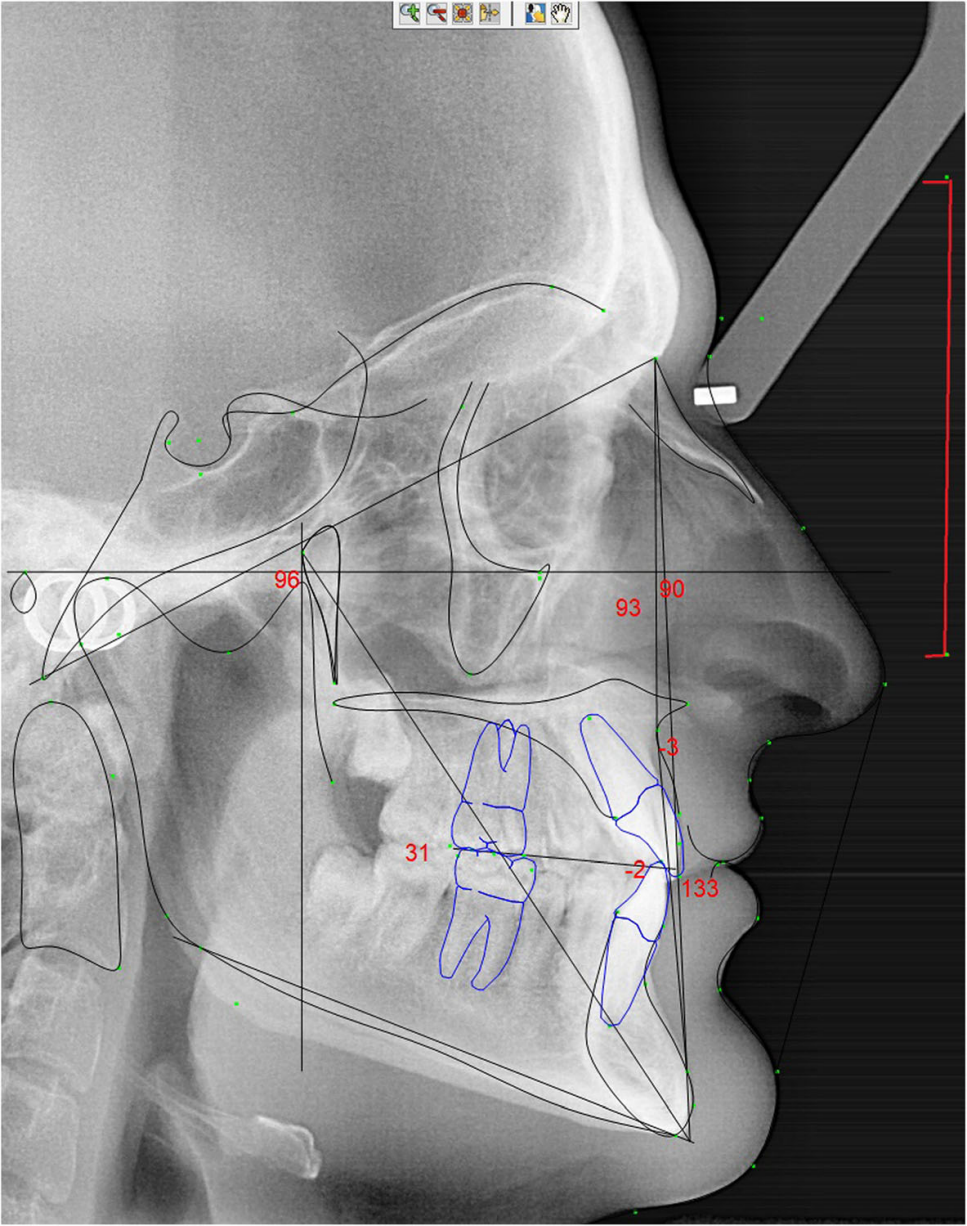
Rickett’s cephalometric dots and plans of an instrumentalist

To ensure intraexaminer reliability, all measurements were repeated in a number of cases by doing again the analysis. For each lateral cephalogram it was determined six cephalometric parameters following the Rickett’s analysis (Fig. 2 and Table 1):

**Table 1:** Linear and angular measurements following the Rickett’s cephalometric analysis

Group/Measurement	Value	Norm	Std Dev	Dev Norm
CRANIOFACIAL RELATION – Cranial Structure				
Cranial Length (mm)	84.6	62.5	2.5	8.8******
Posterior Facial Height (GO-CF) (mm)	81.5	54.8	3.3	8.1******
Cranial Deflection (°)	27.5	27.3	3.0	0.1
Porion Location (mm)	− 58.1	−38.6	2.2	−8.9******
Ramus Position (°)	72.7	76.0	3.0	− 1.1*
CRANIOFACIAL RELATION – Mx Position				
Maxillary Depth (FH-NA) (°)	90.3	90.0	3.0	0.1
Maxillary Height (N-CF-A) (°)	55.1	56.8	3.0	−0.5
SN-Palatal Plane (°)	10.2	7.3	3.5	0.8
CRANIOFACIAL RELATION – Md Position				
Facial Angle (FH-NPo) (°)	92.6	89.6	3.0	1.0*
Facial Axis-Ricketts (NaBa-PtGn) (°)	95.8	90.0	3.5	1.7*
FMA (MP-FH) (°)	21.6	22.9	4.5	−0.3
Total Face Height (NaBa-PmXi) (°)	51.8	60.0	3.0	−2.7**
Facial Taper (°)	65.9	68.0	3.5	−0.6
MAXILLO-MANDIBULAR RELATIONSHIPS				
Convexity (A-NPo) (mm)	−3.1	0.1	2.0	−1.6*
Corpus Length (Go-Gn) (mm)	116.5	80.0	4.4	8.3******
Mandibular Arc (°)	37.6	31.7	4.0	1.5*
Lower Face Height (ANS-Xi-Pm) (°)	39.1	45.0	4.0	−1.5*
DENTAL RELATIONSHIPS – Mx Dentition				
U-Incisor Protrusion (U1-Apo) (mm)	1.9	3.5	2.3	−0.7
U1-FH (°)	119.6	111.0	6.0	1.4*
U-Incisor Inclination (U1-Apo) (°)	24.6	28.0	4.0	−0.8
U6-PT Vertical (mm)	30.9	21.0	3.0	3.3***
DENTAL RELATIONSHIPS – Md Dentition				
L1 Protrusion (L1-Apo) (mm)	−1.6	2.0	2.3	−1.6*
L1 to A-Po (°)	22.6	22.0	4.0	0.1
Mand Incisor Extrusion (mm)	1.3	1.2	2.0	0.0
Hinge Axis Angle	83.8	90.0	4.0	−1.66*
DENTAL RELATIONSHIPS – Mx/Md Dentition				
Interincisal Angle (U1-L1) (°)	132.8	130.0	6.0	0.5
Molar Relation (mm)	−1.9	−3.0	1.0	1.1*
Overjet (mm)	4.0	2.5	2.5	0.6
Overbite (mm)	2.6	2.5	2.0	0.0
Occ Plane to FH (°)	5.2	4.7	5.0	0.1
ESTHETIC				
Lower Lip to E-Plane (mm)	−12.7	−2.0	2.0	−5.3****

As greater the number of *, more severe is the deviation from the norm

1) maxilla position with the base of the skull (maxillar depth – angle formed between the N-PtA plane and the Frankfort horizontal plane;

2) mandible position with the base of the skull (facial angle – formed between the facial plane (N-Pog) and the Frankfort horizontal plane);

3) facial type (for this parameter was taken into account the facial axis, facial angle, mandibular plane, lower facial height and mandibular arch);

4) sagittal skeletal relation (facial convexity – linear measured distance of point A to the facial plane (N-Pog) (The standard value considered is 2 mm ± 2 mm. Participants with values between 0 to 4 mm were considered Class I, over 4 mm Class II and less than 0 mm Class III);

5) upper incisor inclination in relation with the maxilla upper mandibular incisor inclination – angle formed between the incisor’s axis and the plane A-Pog);

6) lower incisor inclination in relation with the mandible (lower mandibular incisor inclination – angle formed between the incisor’s axis and the plane A-Pog);

The IBM SPSS Statistics version 24.0 was used to obtain the variables distribution and posteriorly perform a Fischer exact test to compare the wind instrumentalists group with the string instrumentalists group searching for differences in the variable distribution. The null hypothesis (H0) for the present study was "wind and string instrumentalists have equal cephalometric parameters". Thus, the alternative hypothesis (H1) stated wind and string instrumentalists have differences regarding cephalometric parameters.

## Results

The participants were students of the Escola Superior de Música e Artes do Espectáculo (ESMAE), aged between 18 and 31 years old, playing their instrument for a minimum of 10 years and reported playing approximately 4 to 5 h per day, 6 days per week. From 77 participants, 50 participants played wind instruments and 27 played string instruments. The group of wind instrumentalists was composed by 36 male and 14 female participants. On contrary, the group of string instrumentalists were 21 females and 6 male patients.

Table 2 shows the prevalence of the variables studied. Relatively to the maxillary base the majority of the sample had promaxillary (*n* = 54; 70.1%); for the mandible the majority had promandible (*n* = 41; 53.2%), regarding the facial type most of the sample showed to be brachifacial (*n* = 46; 59.7%) and having a skeletal sagittal Class II (*n* = 48; 62.3%). For the upper incisor the majority of the sample showed to have a pro-inclination regarding the osseous base (maxilla) (*n* = 49; 63.6%); and the lower incisor a orthoposition in relation to the osseous base (mandible) (*n* = 32; 41.6%).

**Table 2:** Variables distribution for the study sample regarding the 6 cephalometric parameters studied

	(Sample = 77)	n	%
Maxilla			
	Retromaxillary	2	2.6
	Orthomaxillary	21	27.3
	Promaxillary	54	70.1
Mandibule			
	Retromandible	5	6.5
	Orthomandible	31	40.3
	Promandible	41	53.2
Facial type			
	Dolichofacial	17	22.1
	Mesofacial	14	18.2
	Brachyfacial	46	59.7
Sagittal Skeletal relation			
	Class I	21	27.3
	Class II	48	62.3
	Class III	8	10.4
Upper incisor			
	Retro-positioned	2	2.6
	Orthopositioned	26	33.8
	Pro-positioned	49	63.6
Lower incisor			
	Retro-positioned	23	29.9
	Orthopositioned	32	41.5
	Pro-positioned	22	28.6

Subsequently, it was evaluated the influence of the type of instrument, wind or string, on the orofacial parameters that were revealed from the cephalometric analysis. A Fisher test was used to compare the six variables between wind instrumentalists and the string instrumentalists. No statistically significant differences were found when compared the maxillary sagittal position, the mandibular sagittal position, the facial type, the sagittal skeletal class and the upper central incisor position (*p* = 0.642), (*p* = 0,594), (*p* = 0,280), (*p* = 0,878) and (*p* = 1,000) respectively, Table 3. For the lower central incisor position with the osseous base the comparison showed statistically significant differences (*p* = 0,011), Table 3.

**Table 3:** Crosstable for the six cephalometric parameters following the Rickett’s analysis and Fisher test used to compare the six variables between wind and string instrumentalists

**Maxilla position**	Orthomaxillary	Promaxillary	Retromaxillary	**Total**	**Fisher exact test (p- value)**
**Wind**	15 (30.0%)	34 (68.0%)	1 (2.0%)	50	**0.642**
**String**	6 (22.2%)	20 (74.1%)	1 (3.7%)	27	
**Total**	21	54	2	77	
**Mandible position**	Retromandible	Orthomandible	Promandible		
**Wind**	4 (8.0%)	18 (36.0%)	28 (56.0%)	50	**0.594**
**String**	1 (3.7%)	13 (48.1%)	13 (48.1%)	27	
**Total**	5	31	41	77	
**Facial type**	Dolichofacial	Mesofacial	Brachyfacial		
**Wind**	10 (20.0%)	7 (14.0%)	33 (66.0%)	50	**0.280**
**String**	7 (25.9%)	7 (25.9%)	13 (48.1%)	27	
**Total**	17	14	46	77	
**Sagittal Skeletal class**	Class I	Class II	Class III		
**Wind**	13 (26.0%)	31 (62.0%)	6 (12.0%)	50	**0.878**
**String**	8 (29.6%)	17 (63.0%)	2 (7.4%)	27	
**Total**	21	48	8	77	
**Upper Incisor Inclination**	Retropositioned	Orthopositioned	Propositioned		
**Wind**	1 (2.0%)	17 (34.0%)	32 (64.0%)	50	**1.000**
**String**	1 (3.7%)	9 (33.3%)	17 (63.0%)	27	
**Total**	23	32	22	77	
**Lower Incisor Inclination**	Retropositioned	Orthopositioned	Propositioned		
**Wind**	15 (30.0%)	26 (52.0%)	9 (18.0%)	50	**0.011**
**String**	8 (29.6%)	6 (22.2%)	13 (48.1%)	27	
**Total**	23	32	22	77	

## Discussion

When analysing wind and string instrument players, the occlusion is a basic clinical information of these individuals. Understanding whether the positions adopted by the CCMC of these musicians and the repetitive movements executed under an unbalanced muscular activity that occurs during musical performance can eventually be detrimental to craniofacial morphology, is a thematic that till nowadays has not yet been completely clear.

The cephalograms were taken to 77 musicians, 50 wind instrument players and 27 string instrument players. This technique has been used since 1931, when this diagnostic tool was introduced in orthodontic practice by Broadbent [27]. The lateral cephalometric radiograph intends to measure and assess craniofacial skeletal, dental and soft tissue relationships, classifying the existing malocclusions and discrepancies. The dentofacial deformities of wind and string instrumentalists which can often result in impaired orofacial functioning, were analysed in this study. Cephalometry has been used for decades in orthodontics, as it is an extremely useful diagnostic tool. In orthodontic practice conventional radiograph cephalometry combines linear and angular measurements or indices derived from these measurements [27].

The results of our findings demonstrated that the majority of the sample had promaxillary (*n* = 54; 70.1%), and when subdividing the sample in wind and string instrumentalists it was possible to observe a similar size of 68% and 74% respectively. Regarding the sagittal position of the mandible, there was a higher difference in the percentage of this occurrence between each group. The wind instrumentalists presented 36% of individuals with orthomandible and 56% with promandible, whereas the string instrument players presented uniforme distributions with 48 and 48%, for the orthomandible and promandible. None of these results were statistically significant, nevertheless there is a higher percentage of individuals with promandible in the wind instrumentalist group compared with the string instrument players.

Regarding the facial pattern of these instrument players, it was possible to observe that both groups had higher percentages of brachyfacial individuals. A previous study highlighted that viola and violin players had a higher prevalence of brachyfacial individuals, manifested by smaller facial heights, greater lengths of mandibular corpus than in controls [11], which was possible to confirm in our study, not only for string (13/27) but also for wind instrumentalists (33/50). Concerning to the sagittal skeletal relationship, it was possible to observe that there are very similar values between wind and string instrument players on the sagittal parameter of Class II, with 62 and 63% respectively. Both of these previous parameters did not present statistically significance.

Accordingly to the relationship of the upper incisor or the lower incisor with the maxilla and mandible, respectively, it was possible to verify that there was statistical significance on the last parameter. The string instrumentalist group showed to have a higher prevalence of the lower central incisor orthopositioned in relation to the mandible.

In relation to the eventual issue of the teeth position, more specifically the central incisor and the embouchure of wind instrumentalists it has been emphasized that tooth irregularities have a negative influence on embouchure comfort and performance. Van der Weijden concluded for example that a Class I relationship without malocclusion seems appropriate for every type of wind instrument and the association of these distinct factors to musical performance is still limited [16].

The current investigation is in agreement with these two issues mentioned above, in the sense that eventually a Class I relationship without malocclusion would be more favorable for the practice for any kind of wind instrument, single reed instrument, double reed instrument or brass instrument. However, there is still lack of sustained information regarding the embouchure mechanism of wind instrument players and the orofacial issues, in particular their craniofacial morphology. Therefore the results are compared with the systematic review of van der Weijden, it is possible to report that there isn’t an accordance regarding the fact reported that the more extreme the malocclusion, the greater the interference with wind instrumentalist’s performance and embouchure comfort. Theoretically this is true, but within the sample of our study we could confirm that 66% of the wind instrument players presented a Class II malocclusion. Dividing the sample into groups of each wind instrument is was possible to notice that in the group of 10 trumpet players, 6 musicians presented a Class II malocclusion, comparing to the group of 10 clarinet players where 8 musicians presented also a Class II malocclusion.

Nevertheless, even with this prevalence of Class II malocclusion within the clarinet players, there could be a dolichofacial pattern associated to counter clockwise mandibular rotation that occurs during the embouchure mechanism. This could provide a greater vertical growth in the molar region, since the mouth is maintained in an open position. But what really happens in terms of craniofacial morphology is the opposite, since within our sample of 50 wind instrument players, there are only 20% of musicians with a dolichofacial pattern, where the majority present a brachyfacial pattern 66%.

If one follows the functional and biomechanical reasons to explain the craniofacial morphology of wind instrument players, all brass instrumentalists could eventually have a Class III malocclusion since the embouchure mechanism involves the protrusion of the lower jaw to obtain the most convenient alignment between the upper and lower jaw during the performance. On our sample two trombone players were classified with a Class I malocclusion, two trombone players with a Class II malocclusion, four trompa players with a Class I malocclusion, one tuba player with a Class I malocclusion, three tuba players with a Class II malocclusion, and from the ten individuals that played trumpet only one presented a Class III malocclusion.

The cephalometric measurements of our research do not confirm that playing a wind or string instrument can influence the craniofacial morphology, since the only statically significant result obtained was the position and relation of the lower central incisor with the osseous base. There are many factors that can contribute for the dental inter-arch relationship, in the three planes of space, namely the sagittal inter-arch relationship, the transversal dimension or the development of anterior open bite or overbite. The wind instrument players can play and practice for more than 5 h per day, nevertheless this time can’t be considered as a continuous force in order to produce or stimulate facial growth or its development. Moreover, it is important to highlight, that in both, wind and string groups, a heterogeneity in the number of the different instruments was present. Ideally, the number of each type of instrument should be similar.

In order to be able to affirm that playing an wind instrument may influence the craniofacial morphology of an individual, there would be the need to provide longitudinal studies where the investigation should be targeted to carry out lateral cephalograms and dental casts to young musicians at the age they start playing their musical instrument, which normally occurs around the age of 7–9 years old, and compare with a control group that does not play any kind of musical instrument. These individuals should be followed until the age of 14–15 years old, where all the permanent teeth would be erupted, not taking in consideration the wisdom tooth.

It is known that there are damaging oral habits like thumb sucking, tongue thrusting or finger sucking with significant repercussions on the development of a malocclusion. The clinician should try initially to eliminate these noxious habits by non-invasive means and then implement an orthodontic treatment, if necessary [28]. There can be a triade of factors, like duration of the habit per day, degree, and the intensity of the habit which will be responsible to produce the detrimental and lasting effects [28]. Although the findings in literature have to be carefully evaluated regarding these matters, there is moderate evidence that for example the use of a pacifier can affect the harmonious development of the orofacial structures being associated to an anterior open bite and posterior cross bite [29]. On the other hand, pure breastfeeding for more than 6 months is inversely associated with daily pacifier use and daily pacifier use is positively associated with daily thumb/digit sucking [30].

The findings of this work are in accordance to the research carried out by Rindischeber et al., where the cephalometric analysis of 61 wind instrumentalists showed similar, normal facial morphology within all groups [13]. However, Brattstorm et al., found significant differences between the musicians and the control group [31]. The musicians had a decreased anterior facial height and wider dental arches, being the findings interpreted with an increased orofacial muscle activity and increased intra-oral pressure resulting from wind instrument playing. The matters related to the applied forces by different wind instruments where already measured by Engelman et al. [32], by means of a transducer, where the instrument pressure was less than the pressure exerted during thumb- sucking and higher than swallowing and whistling [33]. Even in 1950, Salzman [33] referred the fact that “the dentist is, by virtue of his knowledge and experience, in a position to help both the experienced wind instrumentalist and the beginner, and indeed, may even advise an intended musician on the choice of a type of wind instrument most suited to his oral and dental anatomy” [33]. So, for more than 60 years there was already a concern regarding the orofacial issues of wind instrument players and dentofacial morphology.

When studying the craniofacial morphology of wind instrumentalists, some crucial aspects should be considered regarding the time of duration of the applied force, the intensity and the direction. For example, a saxophone player will promote more intrusive forces on the lower incisal teeth due to the embouchure since there is a labioretrusion of the lower lip over the central incisors, comparing for example with the lateral forces applied by the mouthpiece of a trompa player on the lower central incisors [34]. Other studies involving the applied forces associated with the aligner activator demonstrated that the invisible orthodontic forces were greater than the optimal force (0.75—1.25 N) for bodily movement of teeth, as suggested by Proffit and Fields [35]. In addition, a study of Clemente et al. 2019 reported a sample of 30 wind instrumentalists, that applied different values of force to the orofacial structures (ranging from 6 g to 325 g) during the embouchure mechanism [36]. Other matters such as hypertonic lips of wind instrumentalists can be developed by the hyperactivity of the orbicular oris during musical performance, which may also be an important factor on the determination of the position of the lower central incisor in relation to the osseous base. Therefore, it can be possible to have an association between the number of string instrumentalists that have the lower incisor protruded and the fact that these instrument players’ do not necessarily have a hyperfunction of the lower lip like wind instrumentalists when promoting the lip seal during the embouchure mechanism. This finding may suggest that wind musicians are more prone to develop an incisal retroinclination and induce incisor crowding. The placement of a fixed retainer bar in the six anterior inferior teeth could be done as preventive measure, similar to what happens at the end of an orthodontic treatment. Lastly, it is important that further studies, with a longitudinal design can be done in this area where dentistry and orthodontics have very much to improve in terms of knowledge in order to completely understand the eventual impact and associated factors in craniofacial morphology when playing a specific wind or string instrument.

## Conclusions

For both groups a higher prevalence of Class II malocclusion, brachyfacial type, promaxillary and a pro-positioned upper incisor was found. Similar percentages of orthomandible and promandible was found in both groups. The only statically significant difference found between both groups was the position and relation of the lower central incisor with the mandible, which was more orthopositioned in the wind instrumentalist group.

Playing a wind instrument may have little dentofacial influence in the characteristics of an individual, on the other hand insignificant and/or none craniofacial influence.

## Data Availability

The datasets used and/or analysed during the current study are available from the corresponding author on reasonable request.

## Abbreviation

CCMC: Cranio-cervical-mandibular-complex
